# Spatial and Temporal Evolution of Land Use and the Response of Habitat Quality in Wusu, China

**DOI:** 10.3390/ijerph20010361

**Published:** 2022-12-26

**Authors:** Yiming Wei, Hongwei Wang, Mengqi Xue, Yucong Yin, Tiantian Qian, Fangrui Yu

**Affiliations:** 1 College of Geography and Remote Sensing Sciences, Xinjiang University, Urumqi 830017, China; 2 Xinjiang Key Laboratory of Oasis Ecology, Xinjiang University, Urumqi 830017, China

**Keywords:** land use, InVEST model, habitat quality, spatial and temporal evolution, Wusu city

## Abstract

Understanding land use change and its impact on habitat quality (HQ) is conducive to land use management and ecological protection. We used the InVEST model and the GeoDetector model to explore the land use and HQ of Wusu from 1980 to 2020. We found that the spatial distribution of land use in Wusu had the most dramatic change from 2000 to 2010, and accordingly, the habitat quality deteriorated seriously from 2000 to 2010. Via correlation analysis, the response of HQ to land use change is obvious, among which the negative effect of forest land to construction land is the largest, and the positive effect of construction land to water is the largest. However, the overall HQ had the largest negative response to the change of grassland to arable land, and the largest positive response to the change of unused land to grassland. Of the driving factors that cause land use change and thus affect HQ, the human factors are the strongest, and the negative impact on HQ is more irreversible. This study can provide a scientific basis for land use management and ecological protection in Wusu, and can help to further promote the exploration of human activities and ecological responses in arid and semi-arid areas.

## 1. Introduction

According to the 2016 Millennium Ecosystem Assessment, over the past 50 years, ecosystems have changed faster and more extensively than at any other time in human history, and human activities are depleting the earth’s natural capital and putting tremendous stress on the environment [1]. As the core region of the Silk Road Economic Belt, Xinjiang is an important energy base and a typical ecologically fragile area in China and even Central Asia [2]. With the rapid development of economy and society, Xinjiang is now facing the contradiction between energy exploitation, rapid urbanization, and the ecological environment [3,4]. During the period from 2004 to 2018, the economic development of Xinjiang caused a shortage of resources and environmental pollution, but economic development and environmental protection should not be separated, but unified [5]. Although environmental protection will affect economic development in the short term, their relevance will be higher and higher in the context of coordinated development, and environmental protection and economic development can maintain a coordinated situation [6,7].

People’s production and living activities have carried out large-scale transformation of land, including encroachment on ecological space [8]. As a direct indicator of economic growth, land use change has a strong correlation with economic development [9], and it seriously affects the sustainable development of the ecological environment and biological habitat quality [10,11]. HQ is an important function of ecosystem services, which is used to analyze the sustainable development level of biology and environment in the ecosystem [12]. To some extent, HQ can reflect regional biodiversity and ecosystem health, and helps to maintain the biodiversity [13,14]. The land use change dominated by human activities interferes with and changes the style and pattern of the habitat, leading to the decline of landscape connectivity and the intensification of land fragmentation, ultimately affecting material circulation and energy flow within the habitat, and even causing its disappearance [15,16,17]. Therefore, understanding land use change and its impact on HQ is conducive to land use management and biodiversity conservation.

In recent years, dramatic changes in land use/cover have gradually become one of the major threats to global ecosystem maintenance [18]. The transformation of land by human activities has led to the expansion of cultivated land and construction land, serious damage to the original habitat, and even the loss of ecosystem functions and services [19,20]. To explore the extent of habitat change, scholars all over the world have carried out research on HQ, with a view to obtaining data support for ecosystem protection and optimization through quantitative analysis. The previous studies can be roughly divided into two categories according to the content. First, the studies focused on the HQ of specific species, such as the fish habitat of the Zengwen River [21], the marsh rabbit habitat of the hardwood forest in the lower reaches of the Mississippi River in the United States [22], and the mustelid habitat in Iran [23]. They analyzed the HQ suitable for the survival of specific species and tried to deduce the suitability of the entire habitat. The second is to measure the overall HQ, which can be roughly divided into two methods. One of them is to calculate the HQ of the whole region by building a habitat-related indicator system and measuring the habitat parameters of the sample area on the spot. For example, Cecília G. Leal collected field habitat and landscape data from 99 sampling points in 2 regions in the eastern part of the Amazon, Brazil, and determined the relative importance of changes in response variables of the inner flow habitat in the Amazon River basin [24]. Kaire Torn developed an assessment system to assess the structure and function of three important marine habitat types, including sandbars, mudflats, and coral reefs, in the Estonian waters [25]. Thekkendavida Velloth Rehitha conducted seasonal sampling on the Koqin estuary and assessed the ecological HQ of the study area by using the sensitivity of macrobenthos to disturbance. The other method is to use land use data and other threats to evaluate different land use types, estimate the overall regional HQ using the model, and explore the intrusion of human activities on the HQ [26]. Feng Tang used the InVEST model and the linear weighted sum method to evaluate the spatiotemporal change characteristics of HQ and the urbanization level along the Beijing Hangzhou Grand Canal and studied the interaction and stress relationship between urbanization and HQ [27]. Xiaoyin Sun used the InVEST HQ model to monitor the temporal and spatial dynamics of the HQ in the Nansihu Lake basin from 1980 to 2015 and analyzed the potential factors affecting the HQ [28]. Shixiong Song took the Hohhot Baotou Ordos Yulin (HBOY) urban agglomeration in China as an example to simulate the future urban expansion and assess the impact on local natural HQ (NHQ) [29]. Yuanyuan Yang selected 10 landscape indicators to study the evolution of LULC in Taihang Mountains of Hebei Province from 1990 to 2020, and then used the InVEST model to evaluate HQ, and quantitatively analyzed the impact of land use change on HQ [30].

Compared with the field survey method, the InVEST model has irreplaceable advantages. The InVEST model can use less data input to obtain relatively considerable ecosystem service function evaluation results [31]. It can not only greatly reduce the consumption of manpower and material resources but can also avoid the problems of inaccurate results caused by an insufficient sampling rate or uneven distribution of sampling points. Predecessors have also carried out a lot of research on driving factors and produced many effective methods. The GeoDetector model can explore the heterogeneity and explanatory power of different factors in space [32] and is widely used in geography and environmental science.

Previous studies focused on the urban agglomeration or typical ecological areas, studying the encroachment of urban development on habitat, or the intrusion of human activities on the ecological core area. There are still two parts of the relevant research in arid areas that are less or not involved. First, there is little research on the driving factors that cause positive and negative changes in HQ. Second, there is little research on driving factors that are supported by other data, especially human factors. Based on this, we studied the changes of land use and HQ in Wusu and the driving factors of HQ changes. The objectives of this paper were to: (1) obtain the temporal and spatial change characteristics of land use and HQ in Wusu City, (2) measure the response level of HQ to land use change, and (3) explore the factors that cause the land use changes in areas with strong changes in HQ and thus affect HQ by assuming a series of driving factors and combining with auxiliary data. This study puts forward suggestions on land use optimization for sustainable development of ecosystems, further promoting the development of research on human activities and ecological responses in arid and semi-arid areas.

## 2. Materials and Methods

### 2.1. Study Area

**Figure 1:**
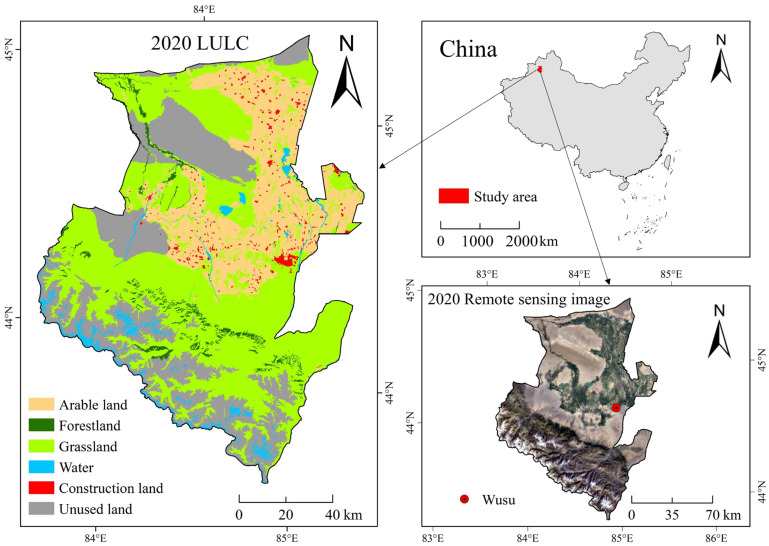
Geographical location of the study area in China and its distribution of LULC (the LULC data of 2020 are from the Resource and Environmental Science Data Center of the Chinese Academy of Sciences, and the remote sensing image of 2020 was taken by the Gaofen-2 resource satellite).

Wusu City (83°20′ E~85°18′ E, 43°26′ N~45°15′ N) is located in the northwest of China (Figure 1). It is a city with rich land use/land cover (LULC) types. It is located in the hinterland of Eurasia continent and has a continental arid climate in the north temperate zone. The daily temperature range is large, the precipitation is rare, the light is sufficient, and the heat is rich [33]. The terrain is mainly high in the south and low in the north. The grassland in the city is the most widely distributed. The construction land and cultivated land are mainly distributed in the northern plains, and the forest land is mainly distributed in the southern mountains. There are rivers such as Kuitun River and Guertu River in the territory.

Wusu City is an important industrial and agricultural production base in Xinjiang, with a GDP of 21.418 billion yuan in 2020, an increase of 4.7% over the previous year. Wusu City is also an important ecological conservation base in Xinjiang, with many natural scenic spots and national forest parks. Since 1980, the population of Wusu City has significantly increased, and the economy has rapidly developed. As an important space for human activities, construction land and arable land have also rapidly expanded, occupying the original ecological space. With the progress of poverty alleviation in China, Wusu City has been lifted out of poverty, and the living standard of residents has been greatly improved. However, there is still a long way to go in tackling ecological and environmental problems.

### 2.2. Data Resources

The land use data, average annual temperature, average annual precipitation, population density, and average ground GDP in 1980, 1990, 2000, 2010, and 2020 are from the Resource and Environmental Science Data Center of the Chinese Academy of Sciences (www.resdc.cn, accessed on 17 September 2022). The soil water content data are from the National Earth System Science Data Center (www.geodata.cn, accessed on 17 September 2022). The data of railways, expressways, national roads, provincial roads, and county roads in the threat factors required by the InVEST HQ Model are from Wusu Natural Resources Bureau and Gaode Map. The economic and social data required in the discussion are from China County Statistical Yearbook in 2001 and 2011. The grid data above are all 100 m resolution, except for the land use data, which is 30 m resolution. In practical operation, the resampling method was used to unify the resolution.

### 2.3. Methods

#### 2.3.1. Land Use Transfer Matrix

By referring to the previous studies [34,35], the land use types in the study area were divided into six types: arable land, forest land, grassland, water, construction land, and unused land. The matrix can reflect the quantitative relationship between land use types in a certain period in terms of time conversion, reflecting the dynamic process of land use change and the change direction guided by human activities [36]. We used the Intersect tool in ArcGIS10.7 software to calculate the cross area between the two datasets, count the cross-conversion area of each land type, and build the matrix [37]. The selected periods in the transfer matrix of this study are: 1980–1989, 1990–1999, 2000–2009, 2010–2020, and 1980–2020.

#### 2.3.2. InVEST Model

The InVEST model was jointly developed by Stanford University, the Nature Conservancy, and the World Animal Foundation in 2007 [38]. The model combines GIS and RS technology, and based on land use/land cover data, quantitatively evaluates ecosystem service functions and carries out spatial visualization [39]. The InVEST model evaluates the impact of threat sources on habitats according to the relative sensitivity of each habitat type to different threat factors and the distance between habitats and threat sources. The impact of threat sources on habitat attenuates with the increase of distance between them, and the attenuation modes include linear and exponential [38,40]. The HQ value obtained by the InVEST model is between 0 and 1, and the larger the value is, the better the HQ is, that is, the higher the level of biodiversity is [41]. Different LULC types have different sensitivities to threat sources. This paper refers to previous studies, combined with experts’ experience and the actual situation of the study area [18,28,42,43], and selected seven threat factors, including arable land, construction land, unused land, railway, highway, national road, provincial road, and county road, to determine the maximum impact distance, weight, and decline type of each threat factor (Table 1), and the sensitivity of different land types to threat factors (Table 2).

#### 2.3.3. Assessment of the Response of HQ to Land Use Change

To quantitatively study the relationship between land use and HQ change at the microscale, we constructed a response index (RI) to measure the response level of HQ to land use change in grid cells. The formula for the calculation is as follows:

(1) $$RI_{ij}=\frac{\sum_{1}^{n}\left(Q_{i}-Q_{j}\right)}{n}$$

where *Q_i_* and *Q_j_*, respectively, represent the HQ values corresponding to land use type *i* and type *j*, and *n* represents the number of grids converted from land use type *i* to type *j*. A negative *RI_ij_* value indicates that the response of HQ is negative when land use type *i* is converted to type *j*, and the greater the value, the greater the negative response. A positive *RI_ij_* value indicates that the response of HQ is positive when land use type *i* is converted to type *j*, and the greater the value, the greater the positive response.

To measure the impact of land use change on the overall HQ, we built the HQ change index (HQCI) to characterize the overall change characteristics of HQ caused by land use. The formula for the calculation is as follows:

(2) $$HQCI=CI_{ij}\cdot S_{ij}$$

where *S_ij_* is the area converted from land use type *i* to type *j.* When *HQCI* is negative, the conversion of land use type *i* to type *j* causes negative changes in the overall HQ. When *HQCI* is positive, land use change causes positive changes. The larger the numerical value, the stronger the change of the overall HQ.

#### 2.3.4. GeoDetector

GeoDetector is a tool to detect and utilize spatial heterogeneity, which can find whether there is spatial correlation between the spatial distribution of statistical variables and independent variables [44,45]. For example, in this paper, the HQ was used as the statistical variable *Y*, and the geographic detector can determine whether there is a certain spatial correlation between the interpretation factor *X_i_* and the statistical variable *Y*. The formula is as follows:

(3) $$q=1-\frac{\sum_{h=1}^{L}N_{h}\sigma_{h}^{2}}{N\sigma^{2}}$$

where *q* can reflect the extent to which factor *X* explains the spatial differentiation of variable *Y*. *L* is the classification of variable *Y* or factor *X*, and *N_h_* is the number of cells in layer *h*. *N* is the total number of units, $\sigma_{h}^{2}$ is the variance of variable *Y* in layer *h*, and $\sigma^{2}$ is the variance of variable *Y* in the whole region.

In addition, interaction detection was also used to determine whether the interaction between the two factors increased or decreased the explanatory power of variable *Y*. Interaction detection can be divided into five types of interaction results, including nonlinear attenuation, single-factor nonlinear attenuation, double-factor enhancement, independence, and nonlinear enhancement.

**Figure 2:**
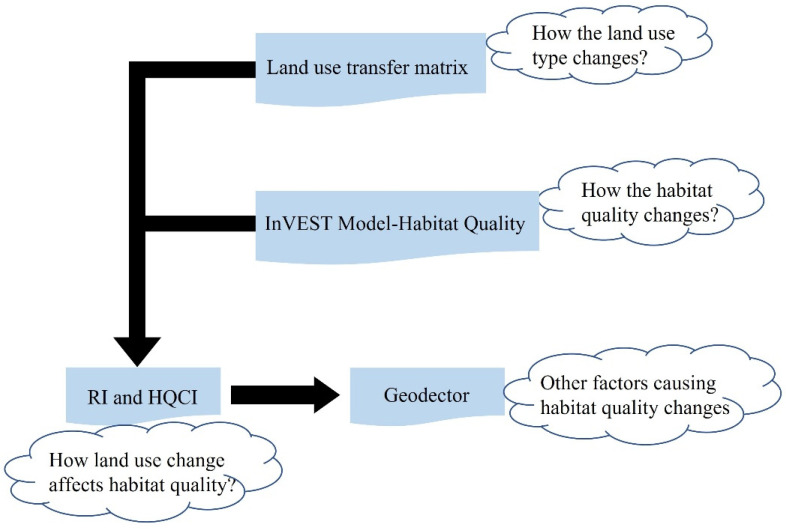
Methods and processes.

The method framework is shown in the figure below(Figure 2):

## 3. Results

### 3.1. Land Use Change Matrix

**Figure 3:**
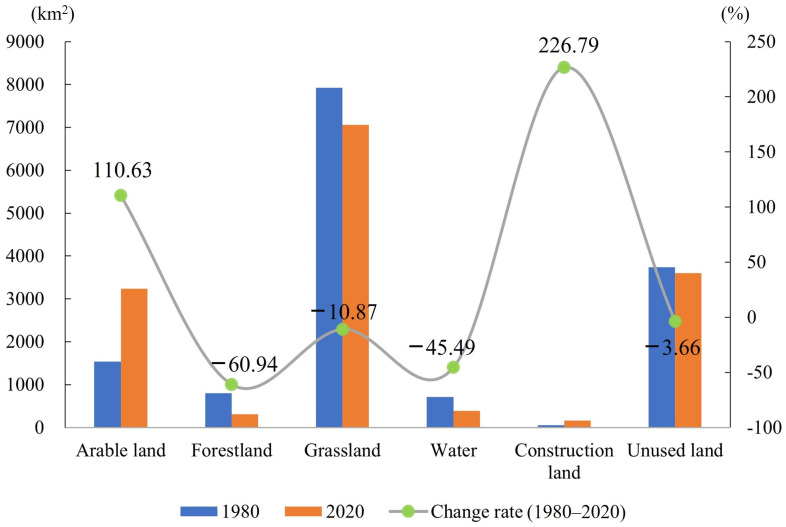
The change of land use from 1980 to 2020.

The land use type of Wusu has a unique structure. The distribution area of grassland was the largest, followed by unused land, and the area of arable land changed the most, increasing by 3237.3891 km^2^ from 1980 to 2020. During those 40 years, the land use structure of Wusu has greatly changed (Figure 3). The areas of forest land, grassland, water, and unused land decreased, and these four types of land gradually flowed into arable land and construction land, resulting in a substantial increase in the area of arable land and construction land. Compared with 1980, the area change rates of arable land and construction land were 110.63% and 226.79%, which was particularly dramatic, and the area change rates of unused land and grassland were the smallest, which were −3.66% and −10.87%.

**Figure 4:**
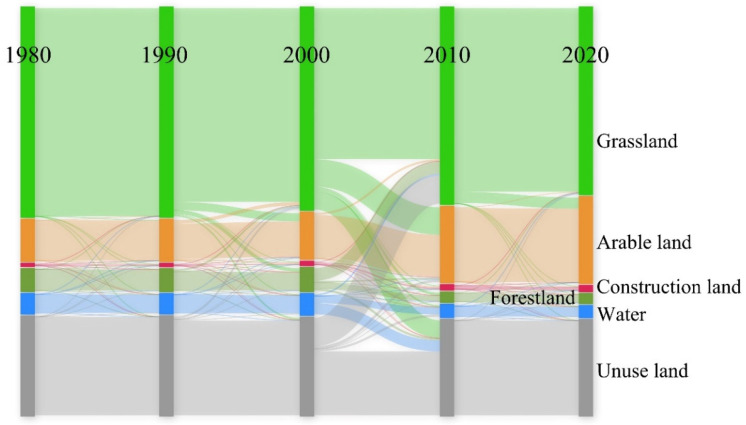
The transfer of land use types.

We built the land use transfer matrix (Table 3) of Wusu from 1980 to 2020 and drew the transfer diagram of land use types in each period (Figure 4). From 1980 to 1990, the change of land use type was weak, mainly due to the outflow of grassland and water. The grassland mainly flows to arable land and unused land, and the water mainly flows to grassland and unused land. From 1990 to 2000, the change of land use types began to increase, and the outflow intensity of grassland was the largest, mainly flowing to arable land and forest land. Arable land and forest land also strongly flowed out. Arable land mainly flows to grassland and construction land, and forest land mainly flows to grassland. During this period, the area of construction land doubled. From 2000 to 2010, land use types changed dramatically, and grassland, forest land, water, and unused land all showed obvious outflow. Grassland mainly flows to arable land and unused land, forest land mainly flows to grassland, water mainly flows to unused land, and unused land mainly flows to grassland. The area of arable land and construction land rapidly expanded during this period, while the area of forest land and water significantly shrank. Due to the complex relationship between inflow and outflow, although grassland and unused land have been strongly transferred, their overall area has not significantly changed. During the period from 2010 to 2020, the main land type change was the transfer of grassland to arable land, followed by the slight expansion of construction land. Other land types had little change and were basically in a stable state.

**Figure 5:**
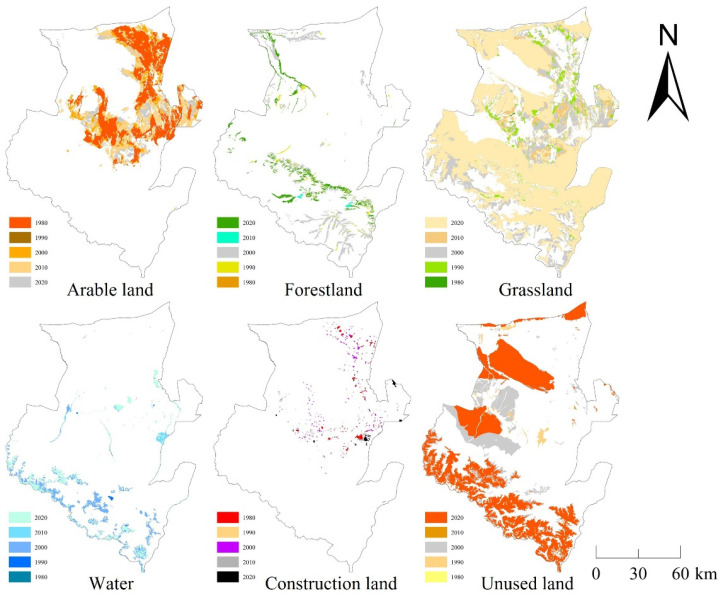
The change of land use areas from 1980 to 2020.

From the perspective of space (Figure 5), the arable land is mainly distributed in the middle and northeast of Wusu, and continuously expanded to the surrounding areas during 1980 to 2020. The expansion of arable land was mainly concentrated in the period from 2000 to 2020, and the expansion area is basically located in the area of grassland degradation. The forest land is mainly located in the south and northwest of Wusu, and the area was shrinking during the study period. The forest land area decreased most seriously from 2000 to 2010, mainly in the north and south of the study area. Grassland is the most widely distributed land use type in Wusu, and the south of the study area is a concentrated grassland. The grassland area in Wusu was continuously reduced, with the most serious situation from 1990 to 2020. In particular, the grassland area in the north has significantly decreased due to the expansion of cultivated land. Water is mainly distributed at the southern boundary of Wusu, mostly ice and snow solid water and its melt water, as well as lakes and rivers in the middle. The area of glaciers in the south has been shrinking in the past 40 years, especially in the period from 2000 to 2010, which is mainly converted into unused land. The construction land is basically distributed in the north of Wusu and was scattered in 1980. During the period from 1990 to 2010, its area sharply increased, and the growth rate exceeded 200%. Unused land is widely distributed in Wusu, and its distribution area is only second to grassland. The types of unused land in the middle and northern low-altitude areas are mainly sandy land, gobi, and saline alkali land, while the types in the southern mountainous areas are mainly bare rock, alpine desert, tundra, etc. With the promotion of the environmental protection policy, the unused land in the north of Wusu decreased in a large area from 2000 to 2010, and gradually turned into grassland.

### 3.2. HQ Change

**Figure 6:**
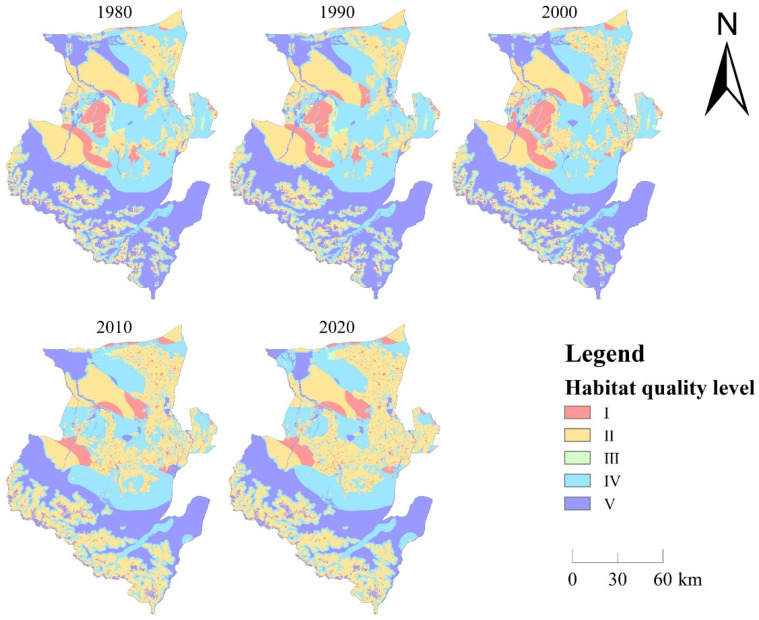
The spatial distribution of HQ of Wusu.

Construction land and arable land, as the types of land most directly affected by human activities, have significantly increased in the past 40 years. Although construction land is the smallest land type in Wusu, its area has increased by more than twice. The substantial increase in the area of construction land and arable land is the result of encroaching on other land types, especially ecological land, which will have irreversible impacts on landscape continuity, biodiversity, and maintenance of ecosystem services, and damage the entire ecosystem. HQ can better reflect the level of biodiversity maintenance and can reflect the ecological quality of a region to a certain extent [12,17]. The HQ module of the InVEST model was used to quantitatively measure the HQ in 1980, 1990, 2000, 2010, and 2020 (Figure 6), and explore the temporal and spatial characteristics of the HQ in Wusu City from 1980 to 2020.

The HQ score range is between 0 and 1. The HQ score is divided into five equal parts, and they are divided into I~V categories from low to high. Category V (0.8~1) represents the best HQ. The HQ in the southern part of Wusu has always been better than that in the northern part, which obtains benefit from the wider distribution of forest land in the southern mountainous area, complete biodiversity, and better maintenance of the ecosystem, which makes the southern part of the HQ perform better. However, from 2000 to 2010, the HQ of the southern mountainous area was significantly degraded, which affected the overall HQ of Wusu. The HQ of category I was mainly distributed in the central and northern parts of Wusu, and basically remained unchanged from 1980 to 2000. From 2000 to 2010, the area significantly changed. Most of the HQ of category I in the central region was transformed into the category IV, and the HQ was greatly improved. From 1980 to 2000, the HQ of category II was mainly distributed in the northwest of Wusu. During the period from 2000 to 2020, the spatial distribution of category II expanded, mainly in the northeastern, central, and southern parts of Wusu. The HQ of category III accounted for the smallest proportion, and the visible distribution in space was only in the central and eastern parts in 1980 and 1990, and the northern part in 2010 and 2020. It can be found that the HQ of category III in the central and eastern parts during the period from 1990 to 2000 was transformed into category II. However, during the period from 2000 to 2020, the HQ of category III in the north was mainly transferred from the category V. The HQ of category IV was mainly distributed in the central and northeastern parts of Wusu from 1980 to 2000. However, after 2000, the HQ of category IV in the east and the central part rapidly decreased, and was mainly converted into the category II. The HQ of category V was mainly distributed in the south and northwest. During 2000–2010, the area of the category V significantly decreased, especially in the northwest and southern border areas.

**Figure 7:**
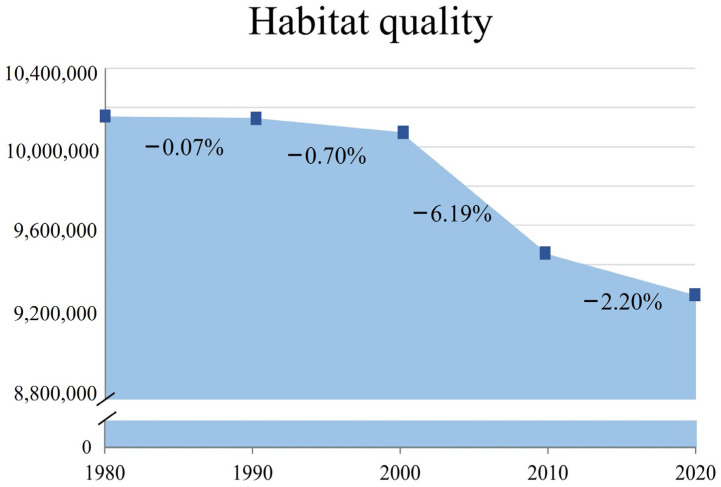
The temporal change in total value of HQ of Wusu.

**Figure 8:**
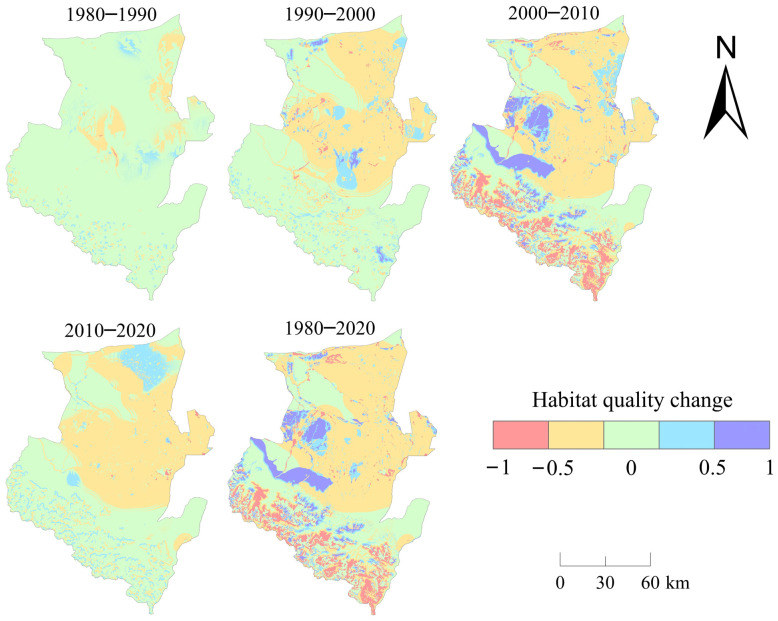
The temporal and spatial changes of HQ of Wusu.

We calculated the total value of HQ in 1980, 1990, 2000, 2010, and 2020, and plotted the change curve (Figure 7) to compare the overall change of HQ in each period. In general, the period with the smallest change in HQ was from 1980 to 1990, and the rate of change in HQ was only −0.07%. The period with the greatest change was from 2000 to 2010, with a rate of change of −6.19%. We performed overlay analysis on the HQ of five periods: 1980~1990, 1990~2000, 2000~2010, 2010~2020, and 1980~2020 (Figure 8). The HQ change was divided into five grades: [−1, −0.5), [−0.5, 0), 0, (0, 0.5], and (0.5, 1], representing negative strong change, negative weak change, no change, positive weak change, and positive strong change, to explore the changes of HQ in Wusu.

From 1980 to 1990, the changes in HQ were relatively small, mostly in the unchanged range. The negative weak changes were mainly concentrated in the central and northeastern parts of Wusu, and the positive weak changes were mainly concentrated in the central and northern parts. There were also scattered positive and negative weak changes in the southern mountainous area. Negative strong changes were distributed in the central area of Wusu, with a small distribution area, and the smallest distribution area was for positive strong changes, whose spatial distribution was almost invisible in the figure. Figure 8 shows that the HQ changed greatly in space from 1980 to 1990, but the change range of the overall HQ value was extremely weak, indicating that the positive and negative changes reached a complementary state in value. From 1990 to 2000, the distribution area of positive and negative changes in HQ increased, especially the negative weak changes. The negative weak changes were concentrated in the central and northeastern areas of Wusu, and the positive weak changes were inlaid in the distribution area of the weak negative changes in a mass shape. From 1990 to 2000, the total value of HQ changed in a relatively balanced state, but its spatial changes were strong, and HQ began to decline.

From 2000 to 2010, the changes in the spatial distribution of HQ were significantly intensified, and the areas with no changes were rapidly reduced. The weak changes were the most widely distributed and were still distributed in the central and northeastern regions. The distribution area of positive and negative strong changes has significantly increased. The positive strong changes were mainly distributed in the west of Wusu, and the negative strong changes were mainly distributed in the southern mountainous areas and other sporadic areas. The HQ in 2010 decreased by 6.19% compared with that in 2000. The changes in HQ during this period were the most drastic among the four periods from 1980 to 2020, both numerically and spatially. The area with no change in HQ from 2010 to 2020 was the largest, and the negative weak changes were still focused on the central and northeastern regions. The distribution area of the positive weak change was second to that of the negative weak change, and it was mainly distributed in the northeast of Wusu in a mass form, and the positive and negative weak changes also had sporadic distribution in the southern mountainous area. Negative strong changes were distributed in the eastern part of Wusu City, while positive strong changes showed almost nothing in space. From 1980 to 2020, the most dramatic changes in land use and HQ occurred from 2000 to 2010, indicating that land use change has a certain impact on HQ change.

### 3.3. Response of HQ to Land Use Change

During the period from 1980 to 2020, the changes of land use and HQ were relatively large, especially during the period of 2000 to 2010, where the changes of land use and HQ were intensified. We considered that land use changes have had a certain impact on HQ. We explored the response of HQ to land use change during the 40-year period from 1980 to 2020 and calculated the response index (RI) of HQ per unit grid to land use change. Since the area of conversion from construction land to forest land was 0, the RI of HQ to conversion from construction land to forest land was not counted, and the response index of HQ to land use change in Wusu from 1980 to 2020 was obtained (Table 4).

The positive and negative values of the HQ response index per unit grid represent the positive and negative responses of HQ to land use conversion. The RI results showed that the responses of HQ were all negative when forest land was converted to other land types, while the responses of construction land to other land types were positive. This shows that the forest land has a positive significance for the maintenance of HQ, while the construction land has a negative significance for the maintenance of HQ. In the process of land use change, the largest positive response of HQ was the conversion of construction land to water, with a value of 0.8974, and the smallest response was the conversion of grassland to water, with a value of 0.0994. The largest negative response was the conversion of forest land to construction land, with a value of −0.9996, and the smallest was the conversion of water area to grassland, with a value of −0.1002. The response of HQ in the process of converting construction land to grassland, unused land to water area, grassland to construction land, forest land to unused land, and water to unused land was also relatively strong, and the absolute values all exceeded 0.6.

To study the impact of land use change on the overall HQ of Wusu, we calculated the HQ change index (HQCI) of various land use conversions. The positive and negative values represent the positive and negative impacts of land use change on the overall HQ (Table 5). The results showed that the largest negative change was the conversion of grassland to arable land, with a value of −628.1324, indicating that the conversion of grassland to arable land had the greatest negative impact on the overall HQ. The smallest negative change was the conversion of water to construction land, with a value of −0.0745. The positive change of the conversion of unused land to grassland was the largest, and the HQCI value was 615.3484, indicating that the conversion from unused land to grassland had the greatest positive impact on the overall HQ. The smallest positive change was the conversion of water to forest land, with an HQCI value of 0.0029. Through the analysis, we found that the CI values and HQCI values of various land use conversions were quite different. The positive HQCI of construction land to water was very small, with a value of 0.0573, but the positive RI of its HQ was the largest, with a value of 0.8974. The negative HQCI of water converted to construction land was the smallest, with a value of −0.0745, but its RI was very large, with a value of −0.8996. The main reason for this difference is that although some land type conversions have a great impact on RI, the area of land type conversions was small, resulting in a low overall change in HQ. On the contrary, although the RI value of some land type changes was small, the HQCI value was high due to the large change area.

### 3.4. Impact Factors of HQ in Typical Sample Areas

**Figure 9:**
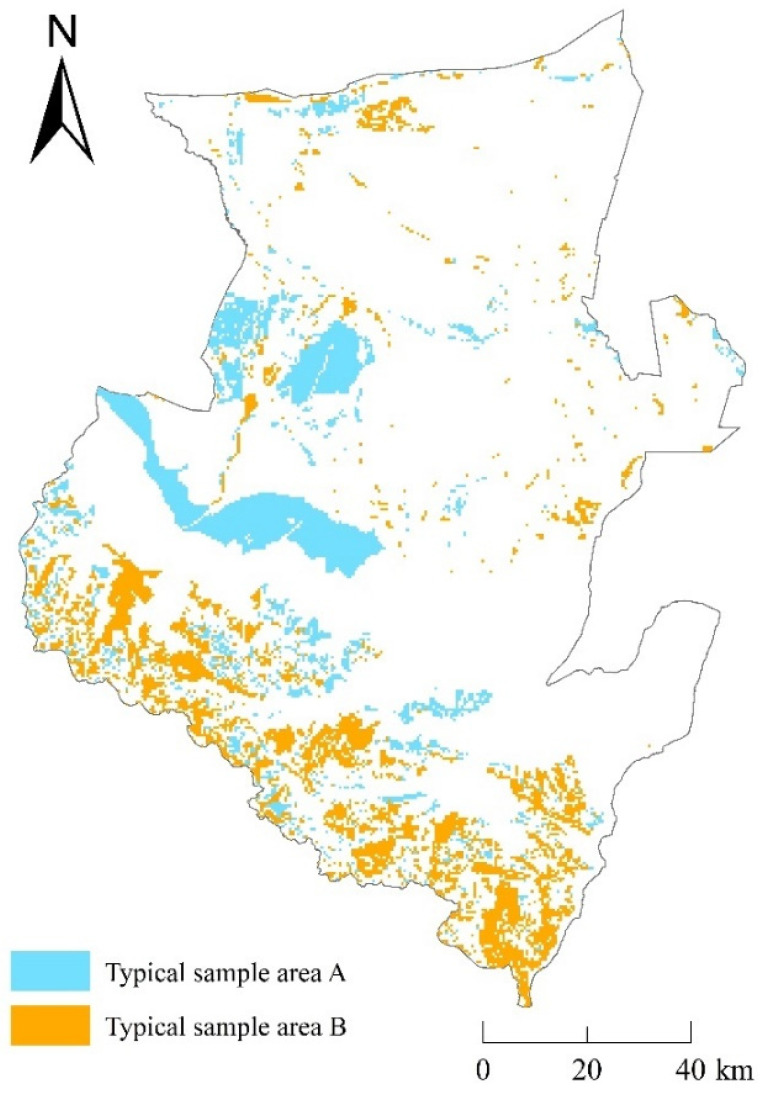
Typical sample areas A and B.

We have quantitatively studied the impact of the overall pattern of land use change in Wusu City on the temporal and spatial changes of HQ, but other factors that cause land use change and thus affect the distribution pattern of HQ have not yet been clarified. Due to the complex transformation of land types in the four periods from 1980 to 2020, it is difficult to accurately quantify the effect of the pattern of change of HQ. However, the changes in land use and the spatial changes of HQ from 2000 to 2010 are basically consistent with the changes from 1980 to 2020. Therefore, we took 2000~2010 as a typical research period. We discussed the main driving factors affecting the spatial changes of HQ by taking the areas with strong positive and negative changes in HQ in Wusu as typical sample area A and typical sample area B (Figure 9). We assumed that natural factors such as annual average temperature, annual precipitation, and soil water content, as well as human factors such as population density and land-average GDP, have driving effects on the strong changes in HQ, and explored other driving factors in areas with strong positive and negative changes in HQ.

**Figure 10:**
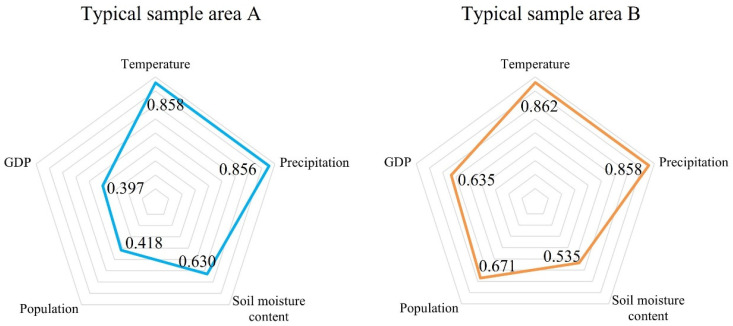
Impact level of factors in typical sample areas.

We used factor detection and cross-detection of geographic detectors to explore other drivers of HQ in typical sample areas A and B, as well as differences in drivers between the areas (Figure 10). The main driving factors of typical sample area A were annual average temperature (q = 0.858) and annual precipitation (q = 0.856), followed by soil water content (q = 0.630). Population density (q = 0.418) and land-average GDP (q = 0.397) had little effect on the positive change of HQ in typical sample area A. The interactions among the five driving factors in typical sample area A were all double-factor enhancement, that is, the q-value score of the interaction between any two driving factors was greater than the largest q-value between them. The main driving factors of typical sample area B were annual average temperature (q = 0.862) and annual average precipitation (q = 0.858), followed by population density (q = 0.671) and land-average GDP (q = 0.635), and soil water content (q = 0.535) had the least impact. The interactions among the five driving factors in typical sample area B were also double-factor enhancement.

However, the *p*-values of the temperature and precipitation of the typical sample areas A and B in the factor detection were both close to 1, indicating that the explanatory power of the temperature and precipitation on the changes in HQ was not significant, that is, the hypothesis that annual temperature and annual precipitation can cause strong changes in HQ does not hold. The effect of soil water content on area A was stronger than that in area B, and it is believed that the improvement of soil water content is beneficial to the improvement of HQ. In addition, the impact of population density and land-average GDP on the strong changes in HQ in area B was significantly stronger than that in area A, indicating that human activities had a stronger impact on negative changes in HQ.

## 4. Discussion

### 4.1. Land Use Change

Generally speaking, land use types usually have obvious differences in topographic gradients [46], and Wusu also follows this law. Forest land is mainly distributed in the southern mountainous areas, and grassland is mainly distributed in the central and southern regions, with slightly lower elevations. Construction land is mainly distributed in the northern and central flat areas, and arable land is basically distributed in the periphery of residential areas, showing a strong dependence on residential areas. Part of the unused land in Wusu is bare rock and bare soil in the southern mountainous area, and the other part is the desert and gobi in the north adjacent to the Gurbantunggut Desert. In addition, there are many rivers and lakes in Wusu, and these water sources ensure the wide distribution of arable land and grassland.

Through previous research, it was found that land use change is related to topography, sunshine hours, and evapotranspiration, and is affected by economic, demographic, and architectural changes [47,48,49,50]. In the previous hypothetical research on driving factors, the impact of natural factors on land use change was not obvious, so here we focused on the driving role of human factors on land use change. Through investigation and literature review, it was found that during the period of 2000~2010, the economy and society of Wusu rapidly developed, and the urban population and rural population both increased by 20,000. The added value of primary production increased from 1185.28 million yuan in 2000 to 1897.72 million yuan in 2010, and the added value of secondary production increased from 541.35 million yuan in 2000 to 4077.55 million yuan in 2010. The rapid development of the primary and secondary industries has brought about the simultaneous growth of the economy and the population. The growth of the population and the economy has greatly increased the demand for residents’ living and food, which has led to a substantial expansion of the construction land and arable land. The expansion of construction land and arable land is bound to encroach on the original ecological land, resulting in the decline of HQ and the gradual weakening of ecosystem services [19,20].

### 4.2. Impact Factors of HQ

In the analysis of driving factors of HQ change, the impact of land use change on HQ is the most intuitive. From the grid unit point of view, the most obvious negative responses are the annexation of construction land on other ecological land, and the degradation of forest land and grassland. In particular, the occupation of ecological space by construction land has accelerated the process of ecological landscape fragmentation, which is almost irreversible damage to ecosystem maintenance [20]. From the overall change of HQ in Wusu, the biggest impact is the transformation of grassland to arable land and unused land. Although the HQ response of the two types of land conversion is not the largest, the high grid response and the large conversion area result in the superposition of quality. Land use change is the most direct spatial characterization factor of HQ change, but not the most fundamental factor. To carry out targeted ecological governance, it is also necessary to clarify the factors that cause land use changes and thus affect HQ changes.

After data analysis, it was found that the typical land type changes in the southern mountainous areas with strong negative changes in HQ from 2000 to 2010 were forest land to grassland and grassland to unused land. After consulting previous reports and investigations, it was found that the economy and society of Wusu rapidly developed from 2000 to 2010, and the demand for wood significantly increased. A large area of forests in the southern mountains have been cut down, tall trees are gradually disappearing, and only herbaceous plants are left on the surface. The grassland surrounding the original forest land has also gradually degraded due to the disappearance of the forest, turning into unused land. This paper considered that the strong negative change of HQ in Wusu City is closely related to anthropogenic deforestation. Due to the difficulty of quantifying deforestation among the elements of human activities, the impact of this factor on HQ has not been quantitatively explored. However, the previous hypothesis found that the explanatory power of annual mean temperature and mean annual precipitation on the strong changes in HQ was not significant, while the negative effects of population density and average GDP on HQ were more obvious than positive ones. This just happened to indirectly confirm that the negative change of HQ in the southern mountainous areas was mainly due to anthropogenic deforestation.

In addition, the main land type transformation of typical sample area A was the conversion of unused land to grassland, which mainly occurred along the Guertu River. Before 2000, the banks of the Gurtu River were severely salinized, with almost no vegetation cover. According to the introduction of the local water management institute, since 2000, the Wusu municipal government has strengthened the management of Liugou Reservoir and repaired the diversion channel from Guertu River to Liugou Reservoir. At the same time, they carried out a series of salinization control work and strengthened the ecological management along the Guertu River. The HQ of the Guertu River Basin in the central and western regions of Wusu has been greatly improved. Regarding the hypothesis of the previous driving factors, the influence of soil water content on the typical sample area A was also verified, and water sources played an important role in the improvement of ecological security [51]. For arid regions, anthropogenic governance has important implications for water quality, water distribution, and water use efficiency [52,53].

### 4.3. The Reasonable Ways of Land Use

The decline of HQ in the southern mountain area of Wusu is due to the degradation of forest land and grassland. Therefore, the local forest and grass department should protect the vegetation ecosystem in the southern mountain area, strengthen supervision, and punish the destruction of forest and grass. At the same time, replanting should be carried out in the vegetation degradation area to restore the original forest grass ecosystem as much as possible, and reasonable grazing control should be carried out. In addition, the government should limit the disorderly expansion of arable land and construction land, especially the large-scale occupation of grassland by cultivated land during 2000–2020 should be resolutely stopped. The future urban housing security of Wusu should focus on intensification, get rid of the previous construction mode of “disorderly planning and low efficiency”, and strengthen the governance of problems such as illegal reconstruction of old urban areas. The planning and construction of new urban areas and industrial parks are strictly prohibited from occupying arable land and grassland. Industrial parks should pay attention to waste disposal to prevent soil pollution from causing vegetation degradation. In recent years, the construction of rural affordable housing in Wusu has been progressing in an orderly manner. The demolition and reclamation of old houses should be put in place to avoid the occupation of arable land for rural residential land, and the occupation of other land to maintain the red line of arable land.

Studies on land governance have been extensively carried out in the Loess Plateau of China, Nigeria, Lebanon, the Mediterranean region, and the southern Amazon, and it is agreed that water resources are an important factor restricting land use management [54,55,56,57,58]. Whether it is the multi-cropping of forest land, grassland, and arable land, or the control of desertification and salinization of unused land, the participation and cooperation of water resources are required. Through analysis, we found that river management measures can effectively improve the soil quality of unused land along the coast and increase the survival rate of vegetation. In the future, the Wusu Municipal Government can further promote the protection and management of water sources such as rivers and lakes, reduce the rate of land desertification and salinization, and carry out the management of unused land through joint methods such as water resource management and vegetation planting. However, the utilization of water resources will also involve the contradiction between the supply and demand of water resources between the production, living, ecological, and other systems in Wusu, the contradiction between the water demand and the supporting potential of water resources, and the maximization of water resources’ utilization efficiency. Therefore, we will further study the management and improvement of land use in the context of water supply and demand in the future.

## 5. Conclusions

Understanding the changes in HQ and its response to land use and other factors is conducive to providing data support and a theoretical basis for the construction of local ecological civilization. The main conclusions of this study were as follows: (1) From 1980 to 2020, the area of arable land increased the most, and the area of grassland decreased the most. The increase rate of construction land area was the largest, and the decrease rate of forest land area was the largest. (2) From 1980 to 2020, the overall performance of HQ in the southern region was better than that in the northern region. The strong positive changes were focused on the central and western regions, and the strong negative changes were mainly distributed in the southern mountainous areas. The changes in HQ were the smallest in 1980–1990 and the largest in 2000–2010. (3) In the process of land use change, the positive response index of HQ was the conversion of construction land to water areas, and the largest negative response index was the conversion of forest land to construction land. In terms of HQCI, the largest positive change was the conversion of unused land to grassland, and the largest negative change was the conversion of grassland to arable land. (4) Among the driving factors that cause land use change and thus affect the strong change of HQ, the anthropogenic factor was the most obvious, followed by soil water content. The impact of human factors on strong negative changes was greater than that of positive strong changes; that is, the damage to HQ caused by human activities is likely to cost more to repair. The results of this study can provide a theoretical basis and scientific guidance for land use and ecological environment protection in Wusu and other similar areas.

## Figures and Tables

**Table 1:** Weights and maximum impact distances of threat factors.

Threat Factor	Maximum Distance/km	Weight	Spatial Decay Type
Arable land	3	0.5	exponential
Construction land	6	0.8	exponential
Unuse land	1	0.4	linear
Railway	6	1	linear
Highway	4	0.8	linear
National road	4	0.8	linear
Provincial road	2	0.5	linear
County road	1	0.4	linear

**Table 2:** The habitat suitability and sensitivity of land use type to each threat factor.

Land Use Type	Habitat Suitability	Threats							
		Arable Land	Construction Land	Unused Land	Railway	Highway	National Road	Provincial Road	County Road
Arable land	0.4	0	0.9	0.4	0.5	0.5	0.4	0.4	0.3
Forest land	1	0.6	0.9	0.5	0.8	0.7	0.6	0.6	0.5
Grassland	0.8	0.5	0.7	0.7	0.6	0.6	0.5	0.4	0.3
Water	0.9	0.6	0.8	0.4	0.7	0.6	0.5	0.4	0.4
Constructionland	0	0	0	0	0	0	0	0	0
Unused land	0.2	0.1	0.1	0	0.2	0.2	0.1	0.1	0.1

**Table 3:** Land use transfer matrix (km^2^).

Time Interval	Land Use Type	Arable Land	Forest Land	Grassland	Water	Construction Land	Unused Land
1980–1990	Arable land	1536.9138	0.0000	0.0585	0.0045	0.0009	0.0117
	Forest land	0.0000	802.6335	0.0702	0.0027	0.0000	0.0198
	Grassland	5.0490	0.0738	7919.8083	0.0387	0.0000	1.4166
	Water	0.0036	0.0009	4.4505	705.3282	0.0000	4.7844
	Construction land	0.0036	0.0000	0.0027	0.0000	49.7277	0.0000
	Unused land	0.0054	0.0108	0.1278	0.1440	0.0000	3740.1453
1990–2000	Arable land	1345.7322	6.2451	127.9926	2.1771	49.6098	10.2186
	Forest land	19.5741	695.3904	66.8124	0.9243	0.7002	19.2141
	Grassland	314.7318	134.8398	7378.4097	31.0887	5.4234	59.8365
	Water	0.9225	0.0216	8.7993	693.2007	0.0018	2.5722
	Construction land	12.4101	0.0000	1.8657	0.0000	35.4528	0.0000
	Unused land	31.1058	7.4412	73.8252	29.1042	1.2663	3603.6351
2000–2010	Arable land	1599.4323	1.3203	73.5633	6.2820	41.6997	2.1789
	Forest land	56.2275	219.4983	478.5507	8.8767	1.2609	79.5267
	Grassland	1045.5948	95.3451	5750.0955	59.1417	31.1562	676.3842
	Water	3.7170	0.1557	57.8862	270.8964	0.0504	423.7902
	Construction land	32.5863	0.0000	2.7477	0.0963	56.7180	0.3060
	Unused land	99.2187	9.8685	1075.2399	83.2446	1.6938	2426.2110
2010–2020	Arable land	2806.2702	0.0243	25.1100	0.0774	5.3433	0.0351
	Forest land	0.0324	311.8581	14.2434	0.0000	0.0000	0.0540
	Grassland	424.1934	1.5786	6989.6592	4.4037	17.2386	1.2078
	Water	0.1359	0.0018	32.8095	384.1875	8.6400	2.7630
	Construction land	2.9214	0.0000	0.1431	0.0000	129.5145	0.0000
	Unused land	3.8367	0.0612	2.4561	0.8532	1.7883	3599.4195
1980–2020	Arable land	1372.0221	1.7658	80.3484	5.0220	75.9942	1.8369
	Forest land	82.1178	176.4603	453.4416	10.1331	2.6379	77.9355
	Grassland	1570.8834	121.7664	5434.7751	57.3597	55.7514	685.8522
	Water	4.1346	0.0288	69.2433	236.5560	0.0828	404.5221
	Construction land	24.7644	0.0000	0.7308	0.0639	23.8680	0.3069
	Unused land	183.4668	13.5027	1025.8758	80.3871	4.1904	2433.0114

**Table 4:** Response index (RI) values for the impact of land use conversions on HQ changes from 1980 to 2020 in Wusu, China.

	Arable Land	Forest Land	Grassland	Water	Construction Land	Unused Land
Arable land	/	0.5998	0.3991	0.4975	−0.4000	−0.2000
Forest land	−0.5999	/	−0.2000	−0.1006	−0.9996	−0.7999
Grassland	−0.3999	0.2000	−0.0001	0.0994	−0.7999	−0.5999
Water	−0.4997	0.1000	−0.1002	−0.0002	−0.8996	−0.6999
Construction land	0.4000	/	0.7990	0.8974	/	0.2000
Unused land	0.2000	0.7998	0.5998	0.6998	−0.2000	/

**Table 5:** HQ change index (HQCI) values of land use conversions from 1980 to 2020 in Wusu, China.

	Arable Land	Forest Land	Grassland	Water	Construction Land	Unused Land
Arable land	−0.0056	1.0590	32.0642	2.4984	−30.3976	−0.3674
Forest land	−49.2608	−0.0012	−90.7008	−1.0197	−2.6369	−62.3418
Grassland	−628.1324	24.3536	−0.5435	5.7018	−44.5937	−411.4382
Water	−2.0660	0.0029	−6.9350	−0.0473	−0.0745	−283.1417
Construction land	9.9056	0	0.5839	0.0573	0	0.0614
Unused land	36.6927	10.8000	615.3484	56.2532	−0.8381	−0.0001

## Data Availability

No new data were created or analyzed in this study. Data sharing is not applicable to this article.
